# Molecular and biochemical changes in *Locusta migratoria* (Orthoptera: Acrididae) infected with *Paranosema locustae*

**DOI:** 10.1093/jisesa/iead077

**Published:** 2023-09-01

**Authors:** Huihui Zhang, Kun Yang, Han Wang, Hui Liu, Wangpeng Shi, Iliya Kabak, Rong Ji, Hongxia Hu

**Affiliations:** International Research Center of Cross-Border Pest Management in Central Asia, Xinjiang Key Laboratory of Special Species Conservation and Regulatory Biology, College of Life Sciences, Xinjiang Normal University, Xinyi Road, Urumqi, Xinjiang Province 830054, China; Tacheng, Research Field (Migratory Biology), Observation and Research Station of Xinjiang, Xinjiang, China; Central for Prevention and Control of Prediction & Forecast Prevention of Locust and Rodent, Xinjiang Uygur Autonomous Region, China; International Research Center of Cross-Border Pest Management in Central Asia, Xinjiang Key Laboratory of Special Species Conservation and Regulatory Biology, College of Life Sciences, Xinjiang Normal University, Xinyi Road, Urumqi, Xinjiang Province 830054, China; Tacheng, Research Field (Migratory Biology), Observation and Research Station of Xinjiang, Xinjiang, China; International Research Center of Cross-Border Pest Management in Central Asia, Xinjiang Key Laboratory of Special Species Conservation and Regulatory Biology, College of Life Sciences, Xinjiang Normal University, Xinyi Road, Urumqi, Xinjiang Province 830054, China; Tacheng, Research Field (Migratory Biology), Observation and Research Station of Xinjiang, Xinjiang, China; College of Plant Protection, China Agricultural University, Beijing, 100193, China; All-Russian Institute of Plant Protection, Sankt-Peterburg, Russia; International Research Center of Cross-Border Pest Management in Central Asia, Xinjiang Key Laboratory of Special Species Conservation and Regulatory Biology, College of Life Sciences, Xinjiang Normal University, Xinyi Road, Urumqi, Xinjiang Province 830054, China; Tacheng, Research Field (Migratory Biology), Observation and Research Station of Xinjiang, Xinjiang, China; International Research Center of Cross-Border Pest Management in Central Asia, Xinjiang Key Laboratory of Special Species Conservation and Regulatory Biology, College of Life Sciences, Xinjiang Normal University, Xinyi Road, Urumqi, Xinjiang Province 830054, China; Tacheng, Research Field (Migratory Biology), Observation and Research Station of Xinjiang, Xinjiang, China

**Keywords:** biological control, energy metabolism, microsporidia, quantitative proteomics

## Abstract

Microsporidia are a group of eukaryotic intracellular parasitic organisms that infect almost all vertebrates and invertebrates. *Paranosema locustae* are specialized parasites of Orthoptera that are often used as biological controls of locusts, with slow effects of action. In this study, we found that after infection with *P. locustae,* changes in energy metabolism in male and female *Locusta migratoria as* were consistent, with no gender differences. During the first 8 days of infection, *L. migratoria* used sugar as a source of energy. After 8 days, lipids and proteins were consumed to provide energy when the spore load was considerably heavy, and energy supply was insufficient. With increasing infection concentration and time, energy conversion from sugar, fats, and proteins was improved, which may explain why high mortality did not occur until about 15 days after *P. locustae* infection. The tandem mass tag-based quantitative proteomics analysis revealed that most altered metabolism-related proteins were upregulated (27 of 29 in the metabolic pathway). This result suggests that *P. locustae* infection accelerated metabolism in *L. migratoria*, which facilitated the pathogen’s life cycle, inhibiting the growth and development of the locusts and eventually killing them. Our findings will be useful to better understand of the chronic pathogenic mechanisms of *P. locustae* and inform on applications of *P. locustae* to control locusts.

## Introduction

Microsporidia are a group of eukaryotic intracellular parasites that infect almost all vertebrates and invertebrates (Li et al. 2018). *Paranosema locustae* is an obligate parasite of Orthoptera. Due to its high spore production potential in hosts, the horizontal and vertical transmission of the disease, and its wide host range within acridid grasshoppers, this parasite can be comprehensively applied to control grasshoppers in grasslands (Shi and Tan 2019). The use of *P. locustae* to control locusts presents environmental advantages over the use of conventional insecticides. This fungal species offers a higher safety profile for humans and other nontarget organisms and allows long-term locust suppression (Henry 1985, Wang 2015). The exact mechanisms by which *P. locustae* causes disease in locusts are unknown. There is a need to determine whether it affects host metabolism in a manner that is similar to other insects, such as silkworms (Hu et al. 2021).

*P. locustae* lack the mitochondria and only contain a mitosome, which is a relic of the mitochondria; some genes involved in energy and metabolism have been lost (Katinka et al. 2001, Williams et al. 2002, Goldberg et al. 2008). All microsporidia have no capacity for the tricarboxylic acid (TCA) cycle, fatty acid oxidation, oxidative phosphorylation, and lack the ATP synthase complex. Some microsporidians, such as *Enterocytozoon bieneusi* also lack the glycolytic pathway (Keeling and Corradi 2011, Timofeev et al. 2020). Therefore, the microsporidia strictly depend on infected host cells for substrates and ATP to complete their proliferation (Cuomo et al. 2012, Dean et al. 2016, Qiang et al. 2019, Timofeev et al. 2020). The most essential energy substrates for insects are soluble proteins, soluble sugars, and fats (Han et al. 2019). We investigated whether *P. locustae* disrupts the normal metabolism of host cells, resulting in the need for the host to consume more energy substrates and synthesize large amounts of ATP to resist microsporidia infection. Microsporidia infection upregulates host energy synthesis and they depend on the energy provided by the host to complete their proliferation and life cycle, resulting in host death (Luo et al. 2021). The microsporidia usually cause chronic infections in hosts, inhibiting their growth and development (Cassal et al. 2020). The *P. locustae* take 10–20 days to induce locust morbidity and mortality (Wang and Guang 1994), during which infection-induced specific metabolic changes in energy substrates and ATP have yet to be established.

The main metabolic pathway of sugar is glycolysis, while lipid synthesis is associated with the tricarboxylic acid cycle. In this study, the key metabolites, that is, glucose-6-phosphate (G6P) (associated with glucose metabolism) and citrate (associated with lipid synthesis), were assessed.

The model organism, *Locusta migratoria* (Orthoptera: Acrididae) (Misof et al. 2014) was used as the host to investigate the utilization of host energy substance by pathogenic. *L. migratoria* were infected with different concentrations of *P. locustae*. After infection, levels of G6P, citrate, total protein, and ATP were measured in locust hemolymph at different time points. The effects of *P. locustae* infection on locust energy substrates were elucidated using its alterations as indicators.

In our previous studies on total protein levels in locust hemolymph after microsporidia infection, electrophoretic profiles of proteins before and after infection were found to be significantly different. This is the first study to use tandem mass tag (TMT) quantitative proteomics techniques to understand these differences. Mass spectrometry-based quantitative analyses of TMT isotope labeling combined with liquid chromatography–mass spectrometry (LC–MS/MS) analysis have become the mainstream technique for studying proteomics and post-translational modifications (Li et al. 2018). Differentially expressed proteins in locust hemolymph before and after *P. locustae* infestation were determined to investigate the changes in *L. migratoria* protein expressions after infection.

Biochemical assays and proteomic analyses will be useful to explore the interactions between pathogenic fungi and hosts, improving our understanding of the pathogenic mechanisms of *P. locustae* and providing a scientific basis for future applications of *P. locustae* in locust control.

## Materials and Methods

### Insect Culture

Migratory locusts used in this study were obtained from laboratory incubation. The populations of *L. migratoria* were reared at 30 ± 2 °C, 50 ± 5% relative humidity, and under a 14:10 h light:dark (L:D) cycle. The locusts were fed daily on fresh wheat shoots and water.

### Experimental *P. locustae*

The *P. locustae* used in this study were acquired from the China Agricultural University.

### Microsporidian Infection

The spores were stored at –20 °C. Third instar nymphs were starved for 12 h. Using a pipette, 5 µl of 1 × 10^4^, 1 × 10^6^, or 1 × 10^8^ spores of *P. locustae* were slowly dripped onto each nymph mouthpart for sucking (Panek et al. 2018). Cages of inoculated and untreated nymphs (controls) were kept under the same conditions as those in locust rearing.

### Collection of Locust Hemolymph

On days 1, 8, and 15 after microsporidia infection, 27 locusts were obtained from 3 concentration gradients for the male, female, and control groups, respectively. Hemolymph (10 µl) was obtained from each locust and mixed with 360 µl of saline. Hemolymph for 9 locusts from the same group was pooled in 1 tube. The hemolymph was collected from the hind legs of locusts. Briefly, the hind legs and surrounding areas were disinfected with 70% alcohol, dried, and 1 hind leg was cut off using scissors. The thorax and abdomen were squeezed, and the hemolymph was quickly drawn and placed in precooled 1.5-ml centrifuge tubes with preadded saline. The samples were immediately mixed in the tubes, and stored at –80 °C for analyses.

### DNA Extraction and Detection by TaqMan Assay

The DNA was extracted from 216 locusts. Experimental locusts were placed in 1.5-ml centrifuge tubes supplemented with 200 μl lysis solution (including 0.2M Tris, 0.3M NaCl, 0.025M EDTANa2, and 0.017M SDS), thoroughly ground and maintained in a water bath at 56 °C for 5 min. Then, the tubes were supplemented with 600 μl of a mixture of phenol, chloroform, as well as isoamyl alcohol and centrifuged at 14,000 rpm for 5 min. The supernatants were discarded. The mixtures were washed with 70% ethanol and centrifuged at 14,000 rpm for 5 min. The ethanol solution was discarded, after which the contents in the tubes were dried and dissolved to extract the DNA by adding 50 μl ddH_2_O. The extracted DNA was measured concentration, and were stored at –20 °C. Finally, the acquired DNA was tested for microsporidia by TaqMan probe assay.

Based on a highly conserved sequence within the *P. locustae* 16S ribosomal RNA gene, a pair of specific primers (forward: 5ʹ-CCGGAGGATCAAAGATGATTAGA-3ʹ; reverse: 5ʹ-CCGTCGG­CATCGTTTACTG-3ʹ) with a product of 59 bp (GenBank accession no. AY305324: 732–674 nt.) was designed using Primer premier 5.0 (PREMIER Biosoft International, Palo Alto, CA). A TaqMan probe (5ʹ-ACCGTCGTAGTTCCG-3ʹ) was designed to fit the *P. locustae* 16S ribosomal RNA gene sequence (GenBank accession no. AY305324: 698-712 nt.), with a FAM (6-carboxy-fluorescein) fluorophore at the 5ʹ end and TAMRA (6-carboxy-tetra­methyl-rhodamine) quencher at the 3ʹ end. Primers and probes were synthesized by Sangon Biotech Shanghai Co. Ltd., China.

For the TaqMan assay, a reaction mixture consisting of 10 μl Platinum Quantitative PCR SuperMix-UDG (catalog no. 11730-017; Invitrogen, America), 0.4 μl (10 μM) of each forward and reverse primer, 0.05 μl (10 μM) of the probe, 0.04 μl (50 nM) of the Rox, and 100 ng of the DNA template was prepared and supplemented with nuclease-free water to 20 μl total volume. A positive control consisting of the DNA of *P. locustae* and a negative control were included in all PCR runs. The TaqMan assay was run in a LightCycler96 Real-Time PCR machine (Roche, Switzerland) under the following conditions: initial denaturation at 95 °C for 2 min followed by 40 cycles of 95 °C for 30 s, 60 °C for 30 s, and 63 °C for 30 s.

### Determination of Energy Materials

In experimental and control groups, concentrations of glucose-6-phosphate dehydrogenase (G6PDH), citrate, and ATP were determined using the Glucose-6-phosphate dehydrogenase (G6PDH) assay kit (catalog no. BC0265; Solarbio, China), citric acid (CA) colorimetric assay kit (catalog no. EBCK351M; Elabscience, China), and the Na^+^K^+^-ATP enzymatic activity assay kit (catalog no. BC0065; Solarbio, China), respectively. Total protein concentrations were determined using the Bradford method (Bradford 1976). Three independent biological replicates were used in this assay, and 3 technical replicates were performed.

### Quantitative Proteomics Analysis

Hemolymph was extracted from *L. migratoria* infected with 10^6^ spores using the same procedure described above, diluted tenfold with 1× PBS buffer and stored in a -80°C freezer until transportation to Applied Protein Technology (Shanghai, China) for TMT quantitative proteomics analysis. The quantitative proteomics has 3 biological replicates.

Functional annotations of proteins were performed using the Blast2GO (https://www.blast2go.com/) program against the nonredundant protein database. The Kyoto Encyclopedia of Genes and Genomes (http://www.genome.jp/kegg/pathway.html) (KEGG) was used to classify and group the identified proteins. Gene Ontology (GO) is an international standardization of the gene function classification system. It has 3 ontologies; molecular functions, cellular components, and biological processes. The KEGG pathway is a collection of manually drawn pathway maps representing our knowledge of molecular interactions and reaction networks.

### Statistical Analysis

Data are expressed as mean ± standard deviations for *n* = 3. Comparisons of means among groups were performed by one-way ANOVA followed by Duncan’s test. *P* ≤ 0.05 was set as the threshold for statistical significance. Analyses were performed using the Statistical Package for Social Sciences, version 26.0 (SPSS, Chicago, USA). Graphs were drawn using PRISM (version 5.00, GraphPad Company, USA).

## Results

### Confirmation of *P. locustae* Infection in *L. migratoria*

Quantitative Cq values (Table 1) showed that all samples were positive at 1, 8, and 15 days postinfection. Copy number of spores in locusts increased with increasing number of infection days and concentration, confirming that *L. migratoria* had been successfully infected with *P. locustae*.

**Table 1.** Results of TaqMan assay after infection of *L. migratoria* with *P. locustae*

Day	Infection concentration	Male	Female	+/–
		Mean ± SD	Mean ± SD	
1	10^4^ spores	31.98 ± 1.56	32.19 ± 1.16	+
	10^6^ spores	33.26 ± 0.82	32.43 ± 0.61	+
	10^8^ spores	30.55 ± 0.73	31.38 ± 0.41	+
	Positive control	15.45 ± 0.06	15.45 ± 0.06	+
	Negative control	—	—	−
8	10^4^ spores	32.32 ± 0.80	33.62 ± 0.73	+
	10^6^ spores	28.22 ± 1.10	28.94 ± 0.67	+
	10^8^ spores	27.66 ± 0.90	28.17 ± 1.24	+
	Positive control	15.74 ± 0.08	15.74 ± 0.08	+
	Negative control	—	—	−
15	10^4^ spores	21.45 ± 0.83	23.78 ± 2.56	+
	10^6^ spores	21.65 ± 1.71	16.16 ± 0.74	+
	10^8^ spores	14.82 ± 0.46	15.62 ± 2.20	+
	Positive control	15.38 ± 0.02	15.38 ± 0.02	+
	Negative control	—	—	−

Standard deviations of means (SD) is shown; “+” represents a positive result; “−” represents a negative result.

### Energy Metabolism After *L. migratoria* Infection With *P. locustae*

#### Changes in G6P levels of *L. migratoria* infection with *P. locustae*

**Fig. 1.**
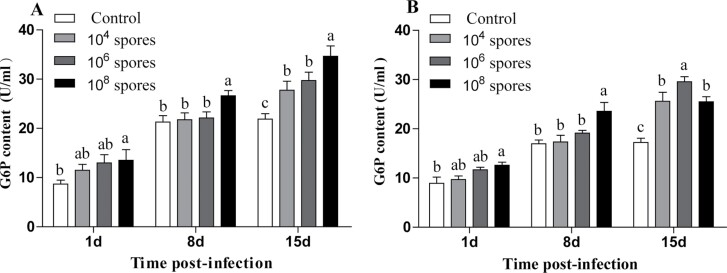
Analysis of changes in G6P levels after infection. A) Male; B) female. Error bars show means ± SD. Values marked with the different letters are significantly different, *P* < 0.05.

Figure 1 shows that male and female *L. migratoria* exhibited consistent changes in G6P levels. The G6P levels in hemolymph gradually increased with increasing infection time. On day 15, the G6P levels were significantly higher than those of the control group (*P* < 0.05).

#### Changes in CA levels of *L. migratoria* infection with *P. locustae*

**Fig. 2.**
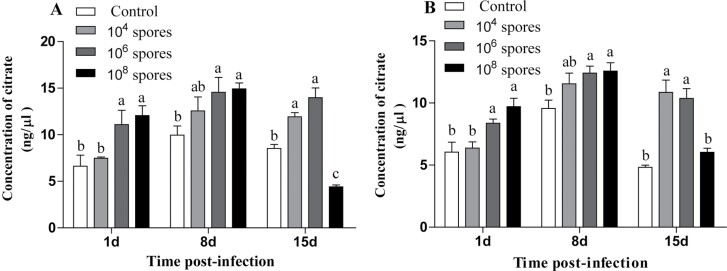
Analysis of changes in citrate levels after infection. A) Male, B) female. Error bars show means ± SD. Values marked with the different letters are significantly different, *P* < 0.05.

Hemolymph samples were collected from *L. migratoria* at different time points after *P. locustae* infection to measure citric acid activities. Figure 2 shows that CA concentrations in hemolymph samples from male and female *L. migratoria* first increased and then decreased. The levels of CA in hemolymph from *L. migratoria* infected with *P. locustae* were significantly higher than those of the control group, particularly on day 15 (*P* < 0.05). In contrast, CA levels in the hemolymph of *L. migratoria* infected with high concentrations of *P. locustae* exhibited a downward trend.

#### Changes in total protein levels of *L. migratoria* infection with *P. locustae*

**Fig. 3.**
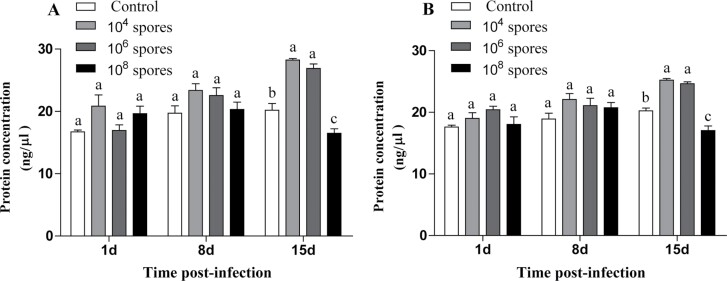
Analysis of changes in total protein levels after infection. A) Male, B) female. Error bars show means ± SD. Values marked with the different letters are significantly different, *P* < 0.05.

Total protein levels in the hemolymph of male and female *L. migratoria* infected with *P. locustae* exhibited the same trend compared to the control group (Fig. 3). On days 1 and 8, differences in protein expressions between the experimental and control groups were insignificant. On day 15, total protein levels in the hemolymph of *L. migratoria* infected with medium and low concentrations of *P. locustae* were significantly higher than those of the control group, while total protein levels in the hemolymph of *L. migratoria* infected with high concentrations of *P. locustae* were significantly lower than those of the control group (*P* < 0.05).

#### Changes in ATP levels of *L. migratoria* infection with *P. locustae*

**Fig. 4.**
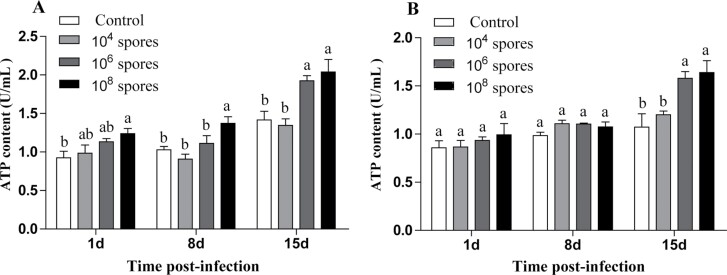
Analysis of changes in ATP levels after infection. A) Male, B) female. Error bars show means ± SD. Values marked with the different letters are significantly different, *P* < 0.05.

The overall change in ATP levels between male and female *L. migratoria* was not significantly different (Fig. 4). On day 15, ATP levels in the hemolymph of *L. migratoria* infected with high concentrations of *P. locustae* were significantly higher than those of the control group (*P* < 0.05).

### Quantitative Proteomics Analysis

#### Protein identification

Interactions between pathogens and hosts are complex as they involve numerous gene regulatory and signaling pathways. To understand the interactions between *L. migratoria* and *P. locustae*, we used TMT as a quantitative proteomic approach to investigate the proteins in *L. migratoria* that were altered following *P. locustae* infection.

A total of 1,506 unique peptides and 266 proteins were detected. Only the proteins with a fold change > 1.2 and a *P*-value < 0.05 were considered to be differently expressed. As a result, 128 proteins were significantly differentially expressed (66 upregulated and 62 downregulated) in *L. migratoria* (Supplementary S. 4).

#### GO enrichment analysis

**Fig. 5.**
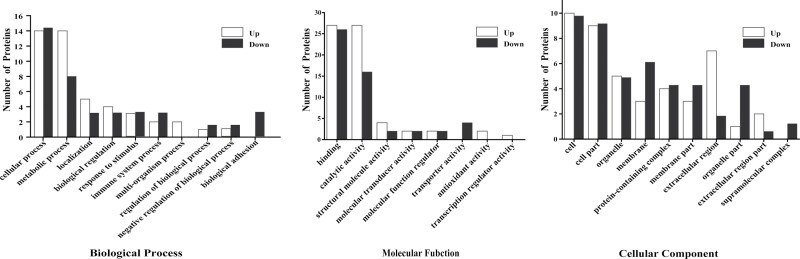
GO categories of upregulated and downregulated proteins. Enrichment of differentially expressed proteins in biological processes, cellular components, and molecular functions was performed by Blast2GO. The number of proteins mapped to GO terms is shown in the left panel.

Biological process-based enrichment analyses of differentially expressed proteins revealed that some GO terms were enriched. Most of the proteins in *L. migratoria* were enriched to cellular and metabolic processes (Fig. 5; Supplementary S. 1). Alterations in metabolic process-related proteins imply that *P. locustae* infection impacts host metabolism.

Molecular function-based enrichment analysis was performed to investigate the potential mechanistic roles that these proteins play in the cell (Fig. 5). Two GO terms, binding and catalytic activity, were highly enriched. The downregulated proteins were enriched in a single category; transcription regulator activity.

Cellular component-based enrichment analysis was performed to investigate the localization of the differentially expressed genes (Fig. 5). Cell and cell part were the most enriched GO terms. Some of the upregulated and downregulated proteins were present in the same categories.

#### KEGG pathway analysis

The KEGG pathway analysis was performed to interpret systemic functions of differentially expressed proteins. In this study, 16 of the proteins were significantly enriched in 5 pathways (*P* < 0.05; Supplementary S. 2), including peroxisome (*P* = 0.015), glycolysis (*P* = 0.035), biosynthesis of nucleotide sugars (*P* = 0.048), HIF-1 signaling pathway (*P* = 0.048), and endocytosis (*P* = 0.048) pathway. Most of the proteins enriched in metabolic pathways were significantly upregulated, while proteins enriched in the protein export pathway were significantly downregulated.

## Discussion

Microsporidia lack the core energy metabolism pathways, therefore, they depend on energy supply from the host for their replication (Pan et al. 2013, Boakye et al. 2017). In cells, sugar is converted into energy through glycolysis and other pathways. The pentose phosphate pathway (PPP) is an integral part of glucose metabolism. Insects convert glucose to glucose-6-phosphate (G6P) (Ge et al. 2020). The G6P is the primary metabolite in glycolysis and serves as a substrate for nucleotide synthesis after oxidization in the pentose phosphate pathway (Luo et al. 2021). In this study, G6P levels significantly increased with time after *P. locustae* infection of both sexes (*P* < 0.05). This was accompanied by increased expressions of 5 proteins involved in glucose metabolism, particularly hexokinase (*P* < 0.05). These results agree with those of . Host glycolysis and TCA cycle can be initiated by microsporidia-secreted hexokinase. Hexokinase induces host biosynthesis to benefit the parasite (Senderskiy et al. 2014, Reinke et al. 2017, Timofeev et al. 2017, Huang et al. 2018), and phosphorylates glucose, which is then absorbed by the parasite (Dolgikh et al. 2019, Luo et al. 2021). The RNA-interfering hexokinase of *N. bombycis* suppresses pathogen proliferation (Huang et al. 2018), suggesting an active glycolytic pathway after *P. locustae* infection, promoting G6P production (Huang et al. 2018). It is corroborative evidence for that the sugar (*P* < 0.05) is an important energy source for *P. Locustae* to proliferate in locusts.

Citrate is involved in the tricarboxylic acid cycle and can activate acetyl-coenzyme A (CoA) carboxylase, the rate-limiting enzyme for the synthesis of fatty acids (Lewis and Majerus 1969). In this study, the lipid metabolism pathway-related proteins, including glycerol-3-phosphate dehydrogenase (NAD^+^), were significantly upregulated. Physiologically, NAD^+^ is mainly involved in glycerophospholipid metabolism. Transcriptome analysis indicated that infection with *N. bombycis* downregulated most of the genes in the long-chain fatty acid synthesis pathway. In contrast, the fatty acid chain degradation pathway significantly upregulated the expressions of thiolytic enzymes (Hu et al. 2021). This shows that microsporidian infection promotes lipid degradation. In this study, citrate levels first increased and then decreased. The experimental results do not match those of proteomic analysis. In contrast with our findings, Hu et al. (2021) infected silkworms with *N. bombycis* and found that host fatty acid levels continuously decreased after infection with microsporidia. These differences were attributed to the different hosts and pathogenic fungi, which may have affected the course of infection. On day 15 after infection with medium-low concentrations of *P. locustae*, citrate levels were significantly elevated (*P* < 0.05), most likely due to high energy requirements for *P. locustae* to proliferate in the host. When the spore load reaches a certain value, citrate is produced, indirectly promoting lipid formation to provide energy. A high number of *P. locustae* progeny were produced on day 15 of the late stage of *P. locustae* infection, leading to insufficient energy supply to host cells and causing lipids to be consumed for energy supply (Li et al. 2018). Therefore, compared with medium-low concentrations, citrate content showed a decrease. After infection with *P. locustae,* hemolymph lipase activities of *L. migratoria* were increased while total fat and hemolymph glycerolipid levels decreased (Chen et al. 2000), which would die after some time and reduce the density of the contemporary locust. This shows the possibility that microsporidia proliferation and microsporidia pathogenicity to locust infection are significantly influenced by lipid metabolism (Franchet et al. 2019).

When the energy provided by fats and sugars is insufficient to meet physiological needs, proteins can be used as energy substrates. A study investigating the relationship between microsporidia infection and host energy metabolism measured the metabolome, including metabolites such as α-ketoglutarate, which is a precursor for the synthesis of amino acids, e.g., glutamic acid as well as proline and found it to be significantly upregulated in the TCA cycle (Luo et al. 2021). The G6P serves as a substrate for nucleotide synthesis after oxidization in the pentose phosphate pathway (Aiston et al. 2004, Ge et al. 2020). We found that G6P levels were significantly upregulated (*P* < 0.05) after *P. locustae* infection. Upregulation of these metabolites promotes the synthesis of nucleotides and amino acids (Williams et al. 2008, Dean et al. 2018). These are accord with the significant increase in total protein levels on day 15 (*P* < 0.05) in the hemolymph of *L. migratoria* infected with medium and low concentrations of *P. locustae*. However, the total protein contents in the hemolymph of *L. migratoria* infected with high concentrations of *P. locustae* were significantly suppressed (*P* < 0.05). This may be due to excess spore loads in the later stages of infection. When the host is low on energy, proteins must be consumed for energy production. Proteomics analyses revealed that protein-associated amino acid metabolism pathways were significantly upregulated. When many spores are present in the locust, and the ATP supply is insufficient, the host consumes proteins to provide energy for the organism to defend against microsporidian infection.

*P. locustae* infection can affect host metabolism. Microsporidia cannot grow or divide outside their host cells. Infection induces metabolic-dependent changes in the host (Dolgikh et al. 1997, Panek et al. 2017). However, how they interact with their hosts and use their resources has not been established. Genomic sequencing of microsporidia revealed that they lack specific metabolic pathways, such as oxidative phosphorylation, electron transport, and the tricarboxylic acid cycle (Luo et al. 2021). Most of the differentially expressed metabolism-related proteins in our study were upregulated (27 of 29 in metabolic pathways), while only 2 were downregulated, supporting the hypothesis that *P. locustae* infection accelerates *L. migratoria* metabolism to benefit the life cycle of the pathogen (Supplementary S. 2).

Proteomics analysis revealed that host energy metabolism-associated proteins were upregulated. *P. locustae* infection promoted glycolysis, fatty acid degradation, and the TCA cycle in the host, which eventually led to increased ATP production (Hoch et al. 2002). Therefore, we determined the ATP concentrations in infected and noninfected *L. migratoria* and found that ATP levels in the hemolymph of *L. migratoria* infected with medium and high concentrations of *P. locustae* were significantly increased on day 15 (*P* < 0.05), consistent with findings from proteomics analysis. However, ATP levels in the low *P. locustae* concentration infected locusts were not significantly increased, presumably because the spore load had not reached the threshold, thus, ATP levels in the host were maintained at the homeostatic equilibrium. This is an important strategy for microsporidia proliferation in host cells over a long period (Luo et al. 2021).

Sugar and lipid levels in the locust hemolymph began to exhibit significant changes on day 1 (*P* < 0.05) after infection with *P. locustae*, whereas total protein levels were significantly increased on day 15 (*P* < 0.05) and changed at different times, suggesting that *P. locustae* are selective in promoting host metabolism (Hu et al. 2021). Regarding the intensity of the content changes, the overall changes in lipid and soluble protein were not as dramatic as those in sugar. The levels of G6P were significantly increased after 15 days (*P* < 0.05) of infection with different *P. locustae* concentrations. It has been reported that microsporidia convert G6P to trehalose during the infection phase, which is the primary carbohydrate source for most of the microsporidian species (Undeen and Solter 1997, Hoch et al. 2002). This implies that during the infection phase, *L. migratoria* first consumes trehalose to provide energy. Lipids and proteins are used as alternative energy supplies when the spore loads are high in the late stages of infection and sugar supply is low. Therefore, ATP levels in the hemolymph of *L. migratoria* were significantly increased (*P* < 0.05) in the later stages of infection because microsporidian invasion increased the metabolic levels of the host.

Female and male *L. migratoria* were separately used in this study because microsporidia exhibit vertical transmission properties. The trends in levels of sugars, lipids, and soluble proteins in male and female locusts after infection were the same, indicating that *P. locustae* infection exerted the same effects on energy substrates in male and female *L. migratoria*. The synthesized energy is initially intended to be used for the growth and development of the host. In a previous study, infection with *N. bombycis* resulted in significant decreases in silkworm body weight and larvae sizes compared to the control group. This indicates that the hosts produce much energy to defend themselves against pathogenic microorganisms, while microsporidia partially absorb and use this energy for their development, causing the hosts to shrink in size. Thus, microsporidia infection significantly impacts host physiology and development (Ma et al. 2013).

In this study, changes in energy metabolism in male and female *L. migratoria* after infection with *P. locustae* were consistent, and there were no gender differences. In the first 8 d of infection, *L. migratoria* used sugar as the energy source. After 8 d when the spore loads were considerably heavy and energy supply was insufficient, lipids and proteins were consumed to provide energy. Energy conversion from sugar, fats, and proteins improved with increasing infection concentration and time. This may be one of the reasons why the high mortality did not occur until about 15 days after *P. locustae* infection. The TMT-based quantitative proteomics analysis revealed that most altered metabolism-related proteins, including hexokinase and glycerol-3-phosphate dehydrogenase (NAD^+^), were upregulated. This indicates that *P. locustae* infection accelerated *L. migratoria* metabolism, which caused the host to produce large amounts of energy to support the proliferation of the pathogen, inhibiting the growth and development of the locusts and eventually causing their death. Our studies on the effect of pathogens on host metabolism may contribute to a better understanding of the chronic pathogenic mechanisms of *P. locustae* and provide a scientific basis for future improvements in *P. locustae* control. However, the specific reason for *P. locustae* long incubation period are unclear and should be further studied.

## Supplementary Material

iead077_suppl_Supplementary_Material
