# Supplementary material for: Molecular and biochemical changes in Locusta migratoria (Orthoptera: Acrididae) infected with Paranosema locustae
Source: J Insect Sci. 2023 Sep 1;23(5):1. doi: 10.1093/jisesa/iead077 (PMC10473453; doi:10.1093/jisesa/iead077)
Supplement: iead077_suppl_Supplementary_Material [file iead077_suppl_supplementary_material.zip › Legends of Supplementary files.docx]

**Legends of Supplementary files:**

Supplementary S1: Date for Figure 5

Supplementary S2: GO categories of up and down regulated proteins

Supplementary S3: Bioinformatic analysis

Supplementary S4: Column and heatmap diagrams
