# Supplementary material for: Molecular and biochemical changes in Locusta migratoria (Orthoptera: Acrididae) infected with Paranosema locustae
Source: J Insect Sci. 2023 Sep 1;23(5):1. doi: 10.1093/jisesa/iead077 (PMC10473453; doi:10.1093/jisesa/iead077)
Supplement: iead077_suppl_Supplementary_Material [file iead077_suppl_supplementary_material.zip › Supplementary S1 Date for Figure 5.docx]

| Type | category | Number of differentially expressed proteins. | |
| --- | --- | --- | --- |
|  |  | Up regulated | Down regulated |
| Biological Process | cellular process | 14 | 9 |
|  | metabolic process | 14 | 5 |
|  | localization | 5 | 2 |
|  | biological regulation | 4 | 2 |
|  | response to stimulus | 3 | 2 |
|  | immune system process | 2 | 2 |
|  | multi-organism process | 2 | 0 |
|  | regulation of biological process | 1 | 1 |
|  | negative regulation of biological process | 1 | 1 |
|  | biological adhesion | 0 | 2 |
|  | developmental process | 0 | 1 |
|  | multicellular organismal process | 0 | 1 |
| Molecular Function | binding | 27 | 26 |
|  | catalytic activity | 27 | 16 |
|  | structural molecule activity | 4 | 2 |
|  | molecular transducer activity | 2 | 2 |
|  | molecular function regulator | 2 | 2 |
|  | transporter activity | 0 | 4 |
|  | antioxidant activity | 2 | 0 |
|  | transcription regulator activity | 1 | 0 |
| Cellular Component | cell | 10 | 16 |
|  | cell part | 9 | 15 |
|  | organelle | 5 | 8 |
|  | membrane | 3 | 10 |
|  | protein-containing complex | 4 | 7 |
|  | membrane part | 3 | 7 |
|  | extracellular region | 7 | 3 |
|  | organelle part | 1 | 7 |
|  | extracellular region part | 2 | 1 |
|  | supramolecular complex | 0 | 2 |

**Table1: GO categories of up and down regulated proteins.**
