# Supplementary material for: Molecular and biochemical changes in Locusta migratoria (Orthoptera: Acrididae) infected with Paranosema locustae
Source: J Insect Sci. 2023 Sep 1;23(5):1. doi: 10.1093/jisesa/iead077 (PMC10473453; doi:10.1093/jisesa/iead077)
Supplement: iead077_suppl_Supplementary_Material [file iead077_suppl_supplementary_material.zip › Supplementary S2 GO categories of up and down regulated proteins.docx]

| Pathway | P value | Pathway ID |
| --- | --- | --- |
| Peroxisome | 0.014754 | ko04146 |
| Glycolysis / Gluconeogenesis | 0.034896 | ko00010 |
| Biosynthesis of nucleotide sugars | 0.048413 | ko01250 |
| HIF-1 signaling pathway | 0.048413 | ko04066 |
| Endocytosis | 0.048413 | ko04144 |

**Table2: KEGG pathway analysis of differentially expressed proteins**
