# Supplementary material for: Molecular and biochemical changes in Locusta migratoria (Orthoptera: Acrididae) infected with Paranosema locustae
Source: J Insect Sci. 2023 Sep 1;23(5):1. doi: 10.1093/jisesa/iead077 (PMC10473453; doi:10.1093/jisesa/iead077)
Supplement: iead077_suppl_Supplementary_Material [file iead077_suppl_supplementary_material.zip › Supplementary S3 Bioinformatic analysis.docx]

Data analysis methods for quantitative proteomic analysis

1. Cluster analysis

Cluster 3.0 (http://bonsai.hgc.jp/~mdehoon/software/cluster/software.htm) and Java Treeview software (http://jtreeview.sourceforge.net) were used for hierarchical clustering analysis. Euclidean distance algorithm for similarity measure and average linkage clustering algorithm (clustering uses the centroids of the observations) for clustering were selected when performing hierarchical clustering. A heat map was used as a visual aid in addition to the dendrogram.

1. Subcellular localization

CELLO (<http://cello.life.nctu.edu.tw/>), which is a multiclass SVM classification system, was used to predict protein subcellular localization.

1. Domain annotation

Protein sequences were searched using the InterProScan software to identify protein domain signatures from the InterPro member database Pfam.

1. GO annotation

Protein sequences of the selected differentially expressed proteins were locally searched using the NCBI BLAST+ client software (ncbi-blast-2.2.28+-win32.exe) and InterProScan to find homologue sequences. Then, gene ontology (GO) terms were mapped and sequences were annotated using the Blast2GO software program. The GO annotation results were plotted by R scripts.

1. KEGG annotation

Following annotation steps, the studied proteins were blasted against the online Kyoto Encyclopedia of Genes and Genomes (KEGG) database (http://geneontology.org/) to retrieve their KEGG orthology identifications and were subsequently mapped to pathways in KEGG.

1. Enrichment analysis

Enrichment analyses were performed by the Fisher’ exact test, considering the whole quantified proteins as the background dataset. Benjamini- Hochberg correction for multiple testing was further applied to adjust the derived p-values. Only functional categories and pathways with p-values under a threshold of 0.05 were considered significant.

1. Protein-protein interaction analysis

The protein–protein interaction (PPI) information of the studied proteins was retrieved from the IntAct molecular interaction database ^[11]^ (http://www.ebi.ac.uk/intact/) by their gene symbols or using the STRING software (http://string-db.org/). Results were downloaded in XGMML format and imported into the Cytoscape software (http://www.cytoscape.org/, version 3.2.1) to visualize and further analyze functional protein-protein interaction networks. The degree for each protein was calculated to assess the importance of the protein in the PPI network.
