# Supplementary material for: Molecular and biochemical changes in Locusta migratoria (Orthoptera: Acrididae) infected with Paranosema locustae
Source: J Insect Sci. 2023 Sep 1;23(5):1. doi: 10.1093/jisesa/iead077 (PMC10473453; doi:10.1093/jisesa/iead077)
Supplement: iead077_suppl_Supplementary_Material [file iead077_suppl_supplementary_material.zip › Supplementary S4 Column and heatmap diagrams.docx]

1. **Column**

**
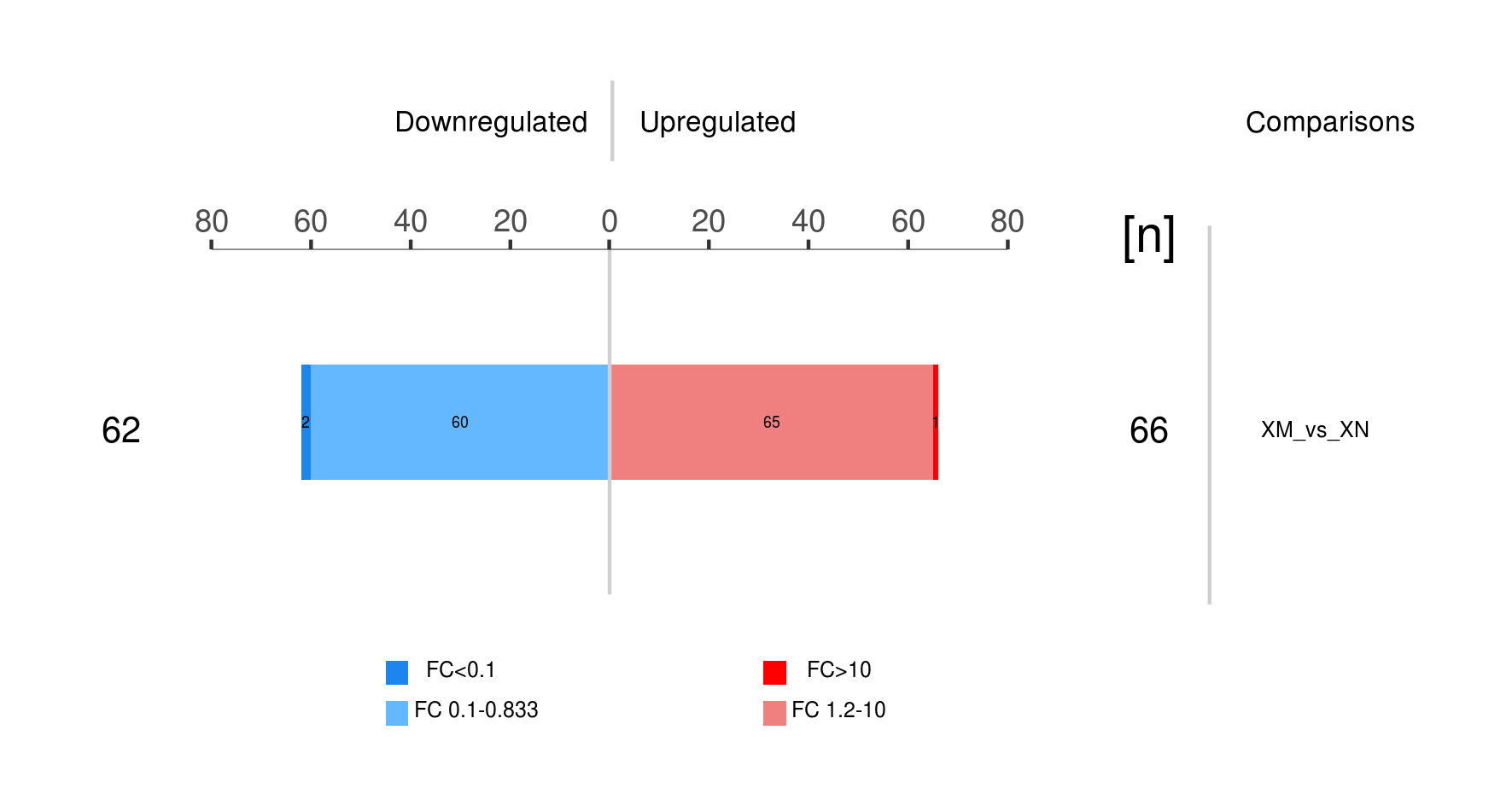
**

**Figure 1: Column of protein expressions after infection with *P. locustae.***

Upregulated: up-regulated differentially expressed proteins.

Downregulated: down-regulated differentially expressed proteins.

1. **Clustering heat map**

**
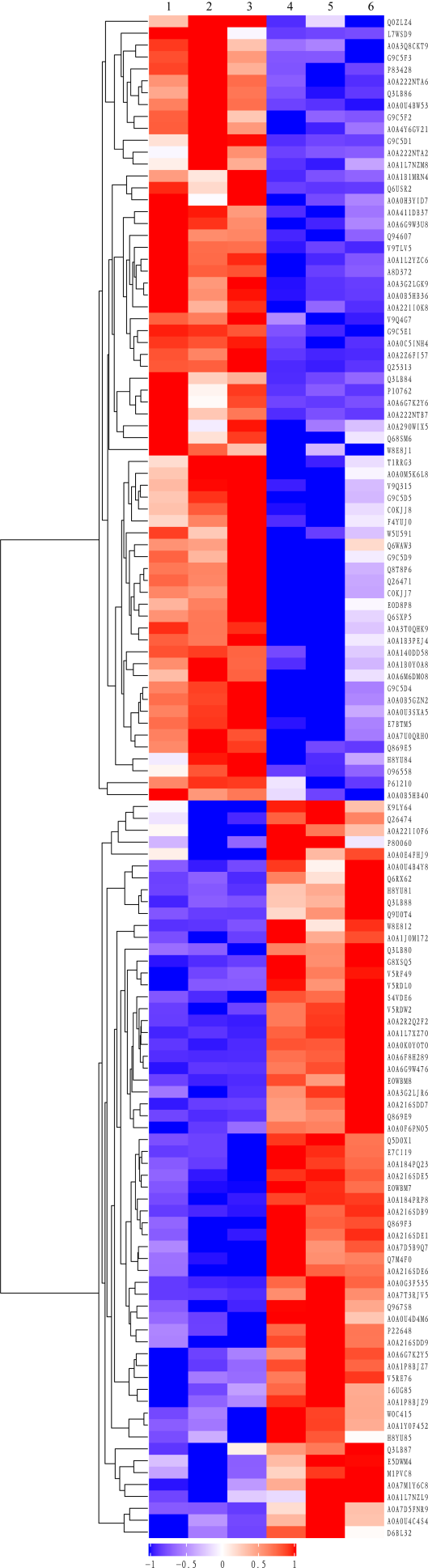
**

**Figure 2: Clustering heat map of expression levels after infection with *P. locustae.***

1-3 represents the infected groups; 4-6 represents the control group; each row represents a protein, in which red represents the protein with up-regulated significance, blue represents the protein with down-regulated significance, and gray does not represent quantitative protein information.
